# Association of LINC00673 Genetic Variants with Progression of Oral Cancer

**DOI:** 10.3390/jpm11060468

**Published:** 2021-05-25

**Authors:** Shih-Chi Su, Chiao-Wen Lin, Po-Chung Ju, Lun-Ching Chang, Chun-Yi Chuang, Yu-Fan Liu, Ming-Ju Hsieh, Shun-Fa Yang

**Affiliations:** 1 Whole-Genome Research Core Laboratory of Human Diseases, Chang Gung Memorial Hospital, Keelung 204, Taiwan; ssu1@cgmh.org.tw; 2 Department of Dermatology, Drug Hypersensitivity Clinical and Research Center, Chang Gung Memorial Hospital, Linkou 333, Taiwan; 3 Cancer Research Laboratory, Xiamen Chang Gung Hospital, Xiamen 361028, China; 4 Institute of Oral Sciences, Chung Shan Medical University, Taichung 402, Taiwan; cwlin@csmu.edu.tw; 5 Department of Dentistry, Chung Shan Medical University Hospital, Taichung 402, Taiwan; 6 School of Medicine, Chung Shan Medical University, Taichung 402, Taiwan; cshy841@csh.org.tw (P.-C.J.); cyi4602@gmail.com (C.-Y.C.); 7 Department of Psychiatry, Chung Shan Medical University Hospital, Taichung 402, Taiwan; 8 Department of Mathematical Sciences, Florida Atlantic University, Boca Raton, FL 33431, USA; changl@fau.edu; 9 Department of Otolaryngology, Chung Shan Medical University Hospital, Taichung 402, Taiwan; 10 Department of Biomedical Sciences, Chung Shan Medical University, Taichung 402, Taiwan; yfliu@csmu.edu.tw; 11 Oral Cancer Research Center, Changhua Christian Hospital, Changhua 500, Taiwan; 12 Graduate Institute of Biomedical Sciences, China Medical University, Taichung 404, Taiwan; 13 Institute of Medicine, Chung Shan Medical University, Taichung 402, Taiwan; 14 Department of Medical Research, Chung Shan Medical University Hospital, Taichung 402, Taiwan

**Keywords:** oral squamous cell carcinoma, long noncoding RNA, single-nucleotide polymorphism, lymph node metastasis

## Abstract

Oral squamous cell carcinoma (OSCC) is a multifactorial malignancy, and its high incidence and mortality rate remain a global public health burden. Polymorphisms in the long intergenic noncoding RNA 673 (*LINC00673*) have been currently connected to the predisposition to various cancer types. The present study attempted to explore the impact of *LINC00673* gene polymorphisms on the risk and progression of OSCC. Three *LINC00673* single-nucleotide polymorphisms (SNPs), including rs11655237, rs9914618, and rs6501551, were evaluated in 1231 OSCCC cases and 1194 cancer-free controls. We did not observe any significant association of three individual SNPs with the risk of OSCC between the case and control group. However, while assessing the clinicopathological parameters, patients carrying at least one minor allele of rs9914618 (GA and AA; OR, 1.286; 95% CI, 1.008–1.642; *p* = 0.043) were found to develop lymph node metastasis more often compared to those who are homozygous for the major allele. Further stratification analyses revealed that this genetic correlation with increased risk of lymphatic spread was further fortified in habitual betel quid chewers (OR, 1.534; 95% CI, 1.160–2.028; *p* = 0.003) or smokers (OR, 1.320; 95% CI, 1.013–1.721; *p* = 0.040). Moreover, through analyzing the dataset from The Cancer Genome Atlas (TCGA), we found that elevated *LINC00673* levels were associated with the development of large tumors in patients with head and neck squamous cell carcinoma and the risk of lymphatic spread in smokers. These data demonstrate a joint effect of *LINC00673* rs9914618 with betel nut chewing or smoking on the progression of oral cancer.

## 1. Introduction

Oral squamous cell carcinoma (OSCC), representing nearly 90% of oral cancer [1], is a neoplasm of multifactorial nature. Various environmental risks intertwine with inherited parameters in the susceptibility to this malignancy [2]. Well-known external risks of OSCC, including human papillomavirus (HPV) infection [3] and habitual use of cigarettes, alcohol, and betel quid [4], have been reported. Moreover, oral carcinogenesis is found to be mediated by a set of genetic alterations that affect programed cell death, cell division, and DNA repair [5]. Nevertheless, the incidence [1] and mortality [6] of OSCC have not improved remarkably in spite of the advance in surgery and other treatment options [7,8] as well as the recognition of risk factors that cause oral malignancies mentioned above [3,4,5,9]. Considering the high heterogeneity and pathogenicity of OSCC, all these cancer risks appear to be mingled and required to evaluate the disease prognosis.

Current advances in sequencing technologies have led to a paradigm shift in our understanding of the possible functions of the noncoding transcriptome [10], in the majority with the focus on the identification of an expanding class of long noncoding RNAs (lncRNAs). To date, an increasing number of lncRNAs is documented to be causally implicated in a variety of human disorders [11], including cancer. Among them, long intergenic noncoding RNA 673 (*LINC00673*), also referred to as SLNCR or SLNCR1, was discovered to be differentially expressed in many tumor types and to promote cancer development and progression through distinct molecular mechanisms [12,13,14,15,16,17,18,19,20,21,22]. Specifically, in spite of being noncoding, transcripts of *LINC00673* can modulate chromatin dynamics and cancer-causing gene expression by serving as a modular scaffold to interact with lysine-specific demethylase 1 (LSD1) and enhancer of zeste homolog 2 (EZH2) [14,16], recruiting androgen receptor (AR) and brain-specific homeobox protein 3a (Brn3a) to facilitate the expression of metalloproteinase 9 (MMP9) [13,21], stabilizing phosphorylation of dishevelled (Dvl) to activate WNT/β-catenin signaling [15], and acting as a competing endogenous RNA (ceRNA) for miR-150-5p [17], miR-515-5p [20], and miR-1231 [12]. These findings collectively indicate that *LINC00673*, behaving as a scaffold, decoy, or signal, can influence cancer biology through genomic targeting, transcriptional regulation, epigenetic mechanisms and antisense interference.

In recent years, genome-wide or targeted gene association studies have revealed a connection of *LINC00673* gene polymorphisms with the risk of many tumor types, such as cancers of the pancreas [12,23], nerve tissues [24,25], stomach [26], cervix [27], and liver [28]. Yet, the impact of *LINC00673* gene polymorphisms on the risk of OSCC remains unclear. Here, we performed a case–control study to investigate the effects of *LINC00673* single nucleotide polymorphisms (SNPs) on the predisposition to oral malignancies.

## 2. Materials and Methods

### 2.1. Subjects

This study encompassed 1231 male cases with OSCC and 1194 cancer-free males, with the approval by the institutional review board of Chung Shan Medical University Hospital in Taichung, Taiwan. Subjects, accrued from 2008 to 2020, provided informed written consent at enrollment. Clinical staging and tumor differentiation of OSCC was determined according to the TNM staging system of the American Joint Committee on Cancer (AJCC) [29] while the disease was first diagnosed. Males without self-reported history of cancer of any site and oral precancerous conditions such as oral submucous fibrosis, verrucous hyperplasia, erythroplakia, leukoplakia, etc., were enrolled to the control cohort. Data concerning age, alcohol consumption, betel nut use, and smoking was recorded for every subject. Betel quid chewing and alcohol drinking are defined as excessive use of betel quid (or related products) and alcoholic drinking, respectively. Smoking is determined by recent use of at least one cigarette per day during the latest three months.

### 2.2. Selection and Genotyping of LINC00673 SNPs

Three common polymorphisms (rs11655237, rs9914618, and rs6501551) from *LINC00673* gene explored in this study were chosen based on their functional potential as predicted by using RegulomeDB [30] (RegulomeDB score < 1). Genomic DNA extraction and genotyping were conducted and analyzed as described previously [31].

### 2.3. Bioinformatics Analysis

The enhancer region bearing rs9914618 in the ENCODE database was visualized by the UCSC genome browser using the hg19 assembly. Prediction of transcription factor binding sites was performed by JASPAR2020 [32].

### 2.4. Statistical Analysis

Significant variation in demographic data between OSCC cases and non-cancer controls was evaluated by using Mann–Whitney U test and Fisher’s exact test. The association of genotypes with OSCC risk was measured using multiple logistic regression methods and adjusted for potential confounders. The differences of *LINC00673* levels in the head and neck squamous cell carcinoma (HNSCC) dataset from The Cancer Genome Atlas (TCGA) were compared by Student’s *t*-test. Data were calculated with SAS statistical software (Version 9.1, 2005; SAS Institute Inc., Cary, NC, USA). A *p*-value < 0.05 was considered significant.

## 3. Results

### 3.1. Cohort Characteristics

In this investigation, 1231 cases with OSCC and 1194 age-matched non-cancer subjects were recruited to evaluate the impact of *LINC00673* SNPs on the development of oral cancer. Only males were enrolled to exclude the potential confounding effect of gender variations on OSCC risk, and cohort characteristics were evaluated (Table 1). Consistent with the findings from others [6,33], significant variations in cigarette smoking, alcohol drinking, and betel quid use were observed between OSCC and control cohort. Among the OSCC cases, lymphatic spread and distal metastasis were observed in 34.7% and 0.9% of patients, respectively.

### 3.2. Association of LINC00673 Gene Polymorphism with the Progression of OSCC

To examine the possible impact of *LINC00673* gene variants on OSCC progression, three SNPs from *LINC00673* gene (rs11655237, rs9914618, and rs6501551) were genotyped in this investigation. The distributions of genotype frequencies for each SNP in our cohort were evaluated (Table 2). No deviation (*p* > 0.05) from Hardy–Weinberg equilibrium in both case and control cohorts was detected for all three SNPs. We failed to individually observe any significant correlation of these *LINC00673* variants with the occurrence of OSCC between the case and control group. Moreover, we investigated the correlations of polymorphic genotypes of *LINC00673* with clinicopathological characteristics of OSCC patients. We found that patients who carry at least one minor allele of rs9914618 (GA and AA; OR, 1.286; 95% CI, 1.008–1.642; *p* = 0.043) were more prone to develop lymph node metastasis as compared with those homologous for the major allele (Table 3). In our stratified analysis, this genetic correlation with increased risk of lymphatic spread was further fortified in patients who were habitual betel quid chewers (OR, 1.534; 95% CI, 1.160–2.028; *p* = 0.003) or smokers (OR, 1.320; 95% CI, 1.013–1.721; *p* = 0.040) (Table 4). These data demonstrate a joint effect of *LINC00673* SNPs and environmental triggers on the progression of oral cancer.

### 3.3. Clinical and Functional Insights of LINC00673 into OSCC

**Figure 1:**
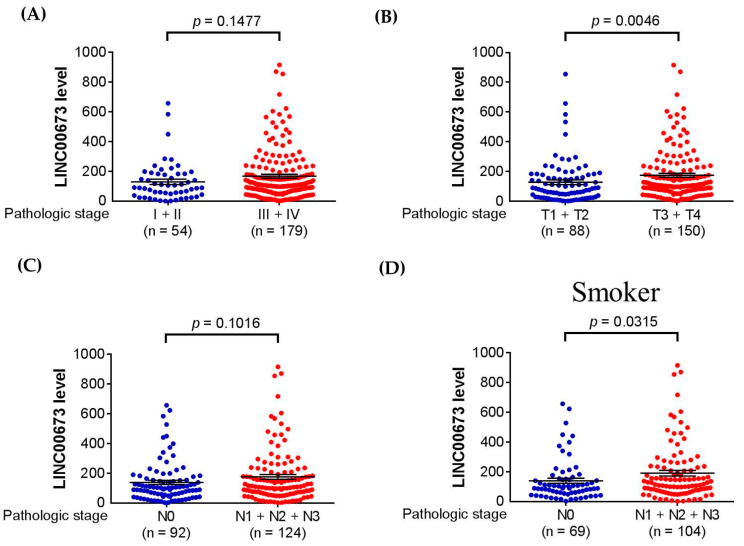
*LINC00673* expression levels are associated with clinicopathological parameters in HNSCC. Correlations of increased *LINC00673* expression with the clinical staging (**A**), tumor size (**B**), and lymph node metastasis (**C**) of HNSCC from The Cancer Genome Atlas (TCGA) database. (**D**) In HNSCC patients who were habitual smokers, increased *LINC00673* expression levels were associated with lymphatic spread. *p* values are calculated with Student’s *t*-test.

**Figure 2:**
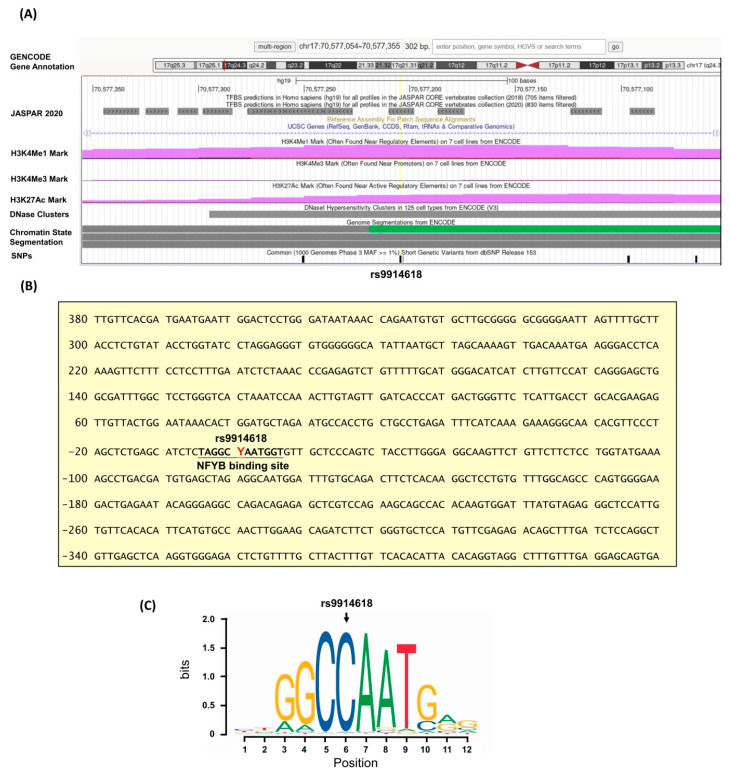
Intron structure of *LINC00673* (NR_137201.2) and the features of rs9914618. (**A**) The intron spanning the chromosome position chr17:70,577,054 to 70,577,355 (reference genome GRCh37.p13) is shown, and the SNPs of *LNC00673* are indicated by the black bars. rs9914628 is centered and marked with reference SNP ID number. The H3K4Me1, H3K4Me3, and H3K27Ac tracks exhibit the enrichment of the mono-methylation of lysine 4, trimethylation of lysine 4, and acetylation of lysine 27 of the H3 histone protein, as determined in ENCODE project. DNase clusters track indicates DNase hypersensitivity regions. Chromatin State Segmentation tracks display chromatin state segmentations by integrating ChIPseq data with a hidden Markov model for H1-hESC embryonic stem cells. The chromatin state region predicted for transcribed regions is highlighted in green. (**B**) Sequence of the intron 1 region. The putative NFYB binding motif (MA0502.2) is shown in bold fonts. Y indicates the position of rs9914628 and denotes C or T. (**C**) Motif logo of NYFB consensus sequences.

As a genetic association of *LINC00673* with oral cancer was noted, additional analyses using public datasets were performed to gain clinical relevance of this gene. We found that higher *LINC00673* expression levels were seen in large tumors in cases with HNSCC in TCGA dataset (Figure 1). Moreover, in HNSCC patients who were habitual smokers, increased *LINC00673* expression levels were associated with lymphatic spread. These data support genetic associations detected in our study and suggest that habitual exposure to environmental risks in combination with altered expression levels of *LINC00673* may affect OSCC progression. Since *LINC00673* SNP, rs9914618, was found to be associated with OSCC progression, we then performed a pilot assessment of the tentative functional relevance of this SNP. We found that rs9914618 is lying on the first intron of the *LINC00673* gene, within an active enhancer region in which multiple epigenetic actions (predominantly H3K4me1) occurred [34] (Figure 2). A further motif search of this enhancer region identified the position of rs9914618 within a putative CCAAT box, a unique pattern of nucleotides present in a high number of the promoter and enhancer regions in eukaryotic genes for the binding with specific transcription factors (e.g., nuclear transcription factor Y, NF-Y) [35,36], suggesting that this intronic variant may be a functional SNP in OSCC progression.

## 4. Discussion

Accumulative evidence has manifested that OSCC progression is a multi-step process orchestrated by both genetic and environmental factors. Here, we reported that *LINC00673* SNP, rs9914618, mediated the invasive potential of OSCC but did not confer the susceptibility to oral malignancies. When comparing only patients who were habitual betel quid or cigarette users, the association of rs9914618 with lymph node metastasis in oral cancer was further strengthened. Our results, for the first time, reveal an interactive effect of rs9914618 with betel nut use or smoking on OSCC progression.

Consistent with our finding that *LINC00673* gene polymorphism was associated with lymphatic spread in oral cancer, the oncogenic role of *LINC00673* in promoting cancer invasion and metastasis through diverse molecular mechanisms has been reported [13,14,15,16,17]. By acting as a scaffold molecule to interact with LSD1, a chromatin modifier that demethylates histone H3 lysine 4 and non-histone targets [37,38], *LINC00673* has been shown to exhibit pro-metastatic properties in gastric cancer [16]. It was also demonstrated that LSD1 functions as a key epigenetic regulator in oral cancer metastasis [39]. Other than LSD1, *LINC00673* facilitated the interaction between DDX3 (DEAD box RNA helicase) and CK1ε (casein kinase 1ε) and thus the phosphorylation of dishevelled, leading to activation of WNT/β-catenin signaling and aggressiveness of lung adenocarcinoma [15]. Our results and findings from others imply that polymorphic transcripts of *LINC00673* may exert differential affinities with distinct binding partners to manage cancer invasiveness and metastasis. In addition to protein molecules, *LINC00673* could sponge miR-150-5p and modulate epithelial-mesenchymal transition in lung cancer [17]. In a recent study aiming to discover specific plasma microRNAs as OSCC biomarkers, miR-150-5p was identified to be useful to monitor malignant progression in oral cancer [40]. These data support the genetic association of *LINC00673* with lymphatic spread observed in our study and reveal a multifaceted role of *LINC00673* in cancer progression via [27].

*LINC00673* SNP, rs11655237, has been extensively reported for its association with the occurrence, progression, and prognosis of multiple tumor types [41]. This genetic variation (G > A) creates a miR-1231–binding site, which diminishes the effect of *LINC00673* and, thus, may confer susceptibility to cancer [12]. In addition to the changes in functionality, the polymorphic allele of rs11655237 was proposed to cause reduced expression of *LINC00673* [27]. Of note, unlike several previous reports linking rs11655237 to cancer risk [12,24,25,26,27,28], we failed to detect a genetic association of rs11655237 with the predisposition to OSCC. This discrepancy may be, in part, accounted for by a relative weak effect of specific SNPs or influence from other potential etiological confounders.

In the present study, we reported that *LINC00673* gene polymorphism, rs9914618, was linked to the invasive potential of OSCC. Our exploratory evaluation of functional relevance demonstrated that rs9914618 is located within a CCAAT box, a putative binding motif of NF-Y (nuclear transcription factor Y) [36] and C/EBPs (CCAAT/enhancer binding proteins) [35]. NF-Y has been emerging as a key transcriptional regulator for many genes overexpressed in several cancer types [42], and a tumor suppressive role for C/EBPs has previously been demonstrated in HNSCC [43]. These findings suggest that rs9914618 polymorphisms may render a distinct expression profile of cancer-related genes mainly through impaired interactions with NF-Y or C/EBPs, thereby mediating OSCC progression.

Our data revealed an influence of *LINC00673* SNPs on the lymph node metastasis of oral cancer; yet, extra work is required to address several limitations in the study. One is that the effects of *LINC00673* gene polymorphisms on the risk of developing OSCC may be underestimated because of a lack of enumerative definition for betel quid consumption, alcohol use, and smoking. Another weakness is that the molecular mechanism underlying the promotive role of rs9914618 in oral cancer metastasis remains an open question. Whether the genetic polymorphism (G > A) controls its expression or alters the binding to its interacting proteins or microRNAs, thereby affecting OSCC biology, requires further investigations. Additionally, RNA is unavailable in situ hybridization analysis of *LINC00673* on neoplasm lesion and/or lymph node metastasis lesion in OSCC. Moreover, the findings reported in the present investigation might be unable to be applied to other ethnic groups unless replication experiments are conducted.

## 5. Conclusions

In conclusion, our results demonstrate an association of *LINC00673* rs9914618 with the metastatic potential in oral cancer. Elevated *LINC00673* expression levels contribute to an inclination to develop large tumors in HNSCC patients and lymph node metastasis in habitual smokers. These findings reveal a novel genetic relationship between *LINC00673* variants and OSCC progression.

## Figures and Tables

**Table 1:** The distributions of demographical characteristics and clinical parameters in 1195 controls and 1125 cases with OSCC.

Variable	Controls (N = 1194)	Patients (N = 1231)	*p*-Value
Age (yrs)	53.90 ± 10.03	55.53 ± 10.85	
<55	565 (47.3%)	574 (46.6%)	*p* = 0.733
≥55	629 (52.7%)	657 (53.4%)	
Betel quid chewing			
No	995 (83.3%)	303 (24.6%)	
Yes	199 (16.7%)	928 (75.4%)	*p* < 0.001 *
Cigarette smoking			
No	558 (46.7%)	193 (15.7%)	
Yes	636 (53.3%)	1038 (84.3%)	*p* < 0.001 *
Alcohol drinking			
No	957 (80.2%)	648 (52.6%)	
Yes	237 (19.8%)	583 (47.4%)	*p* < 0.001 *
Stage			
I + II		575 (46.7%)	
III + IV		656 (53.3%)	
Tumor T status			
T1 + T2		605 (49.1%)	
T3 + T4		626 (50.9%)	
Lymph node status			
N0		804 (65.3%)	
N1 + N2 + N3		427 (34.7%)	
Metastasis			
M0		1220 (99.1%)	
M1		11 (0.9%)	
Cell differentiation			
Well differentiated		174 (14.1%)	
Moderately or poorly differentiated		1057 (85.9%)	

Mann–Whitney U test was used between healthy controls and patients with oral cancer. * *p*-value < 0.05 as statistically significant.

**Table 2:** Odds ratio (OR) and 95% confidence interval (CI) of OSCC associated with *LINC00673* genotypic frequencies.

Variable	Controls (N = 1194) n (%)	Patients (N = 1231) n (%)	OR (95% CI)	AOR (95% CI)
rs11655237				
CC	772 (64.7%)	784 (63.7%)	1.000	1.000
CT	371 (31.1%)	398 (32.3%)	1.056 (0.889–1.256)	1.034 (0.834–1.282)
TT	51 (4.2%)	49 (4.0%)	0.946 (0.631–1.418)	0.675 (0.405–1.125)
CT + TT	422 (35.3%)	447 (36.3%)	1.043 (0.883–1.231)	0.986 (0.802–1.213)
rs9914618				
GG	770 (64.5%)	799 (64.9%)	1.000 (reference)	1.000 (reference)
GA	370 (31.0%)	380 (30.9%)	0.990 (0.832–1.178)	0.954 (0.768–1.184)
AA	54 (4.5%)	52 (4.2%)	0.928 (0.626–1.375)	0.652 (0.398–1.068)
GA + AA	424 (35.5%)	432 (35.1%)	0.982 (0.831–1.160)	0.911 (0.740–1.121)
rs6501551				
AA	890 (74.5%)	919 (74.7%)	1.000 (reference)	1.000 (reference)
AG	279 (23.4%)	294 (23.9%)	1.021 (0.846–1.232)	1.039 (0.822–1.312)
GG	25 (2.1%)	18 (1.4%)	0.697 (0.378–1.287)	0.689 (0.323–1.471)
AG + GG	304 (25.5%)	312 (25.3%)	0.994 (0.828–1.193)	0.911 (0.740–1.121)

The odds ratio (OR) with their 95% confidence intervals were estimated by logistic regression models. The adjusted odds ratio (AOR) with their 95% confidence intervals were estimated by multiple logistic regression models after controlling for betel nut chewing, alcohol and tobacco consumption.

**Table 3:** Clinical statuses and *LINC00673* rs9914618 genotype frequencies in oral cancer.

Variable	*LINC00673* (rs9914618)			
	GG (%) (n = 799)	GA + AA (%) (n = 432)	OR (95% CI)	*p*-Value
Clinical Stage				
Stage I/II	373 (46.7%)	202 (46.8%)	1.00	*p* = 0.980
Stage III/IV	426 (53.3%)	230 (53.2%)	0.997 (0.788–1.261)	
Tumor size				
T1 + T2	393 (49.2%)	212 (49.1%)	1.00	*p* = 0.970
T3 + T4	406 (50.8%)	220 (50.9%)	1.005 (0.795–1.270)	
Lymph node metastasis				
No	538 (67.3%)	266 (61.6%)	1.00	*p* = 0.043
Yes	261 (32.7%)	166 (38.4%)	1.286 (1.008–1.642)	
Distant metastasis				
No	794 (99.4%)	426 (98.6%)	1.00	*p* = 0.175
Yes	5 (0.6%)	6 (1.4%)	2.237 (0.679–7.371)	
Cell differentiation				
well	111 (13.9%)	63 (14.6%)	1.00	*p* = 0.740
Moderate/poor	688 (86.1%)	369 (85.4%)	0.945 (0.677–1.320)	

**Table 4:** Clinical statuses and *LINC00673* rs9914618 genotype frequencies in oral cancer among 928 betel quid chewing and 1038 smoking.

Variable	*LINC00673* (rs9914618)					
	Betel Quid Chewing (n = 928)			Smoking (n = 1038)		
	GG (%) (n = 590)	GA + AA (%) (n = 338)	*p*-Value	GG (%) (n = 672)	GA + AA (%) (n = 366)	*p*-Value
Clinical Stage						
Stage I/II	288 (48.8%)	148 (43.8%)	*p* = 0.140	318 (47.3%)	165 (45.1%)	*p* = 0.489
Stage III/IV	302 (51.2%)	190 (56.2%)		354 (52.7%)	201 (54.9%)	
Tumor size						
≤T2	300 (50.8%)	163 (48.2%)	*p* = 0.442	345 (51.3%)	180 (49.2%)	*p* = 0.506
>T2	290 (49.2%)	175 (51.8%)		327 (48.7%)	186 (50.8%)	
Lymph node metastasis						
No	410 (69.5%)	202 (59.8%)	*p* = 0.003 *^,a^	454 (67.6%)	224 (61.2%)	*p* = 0.040 *^,b^
Yes	180 (30.5%)	136 (40.2%)		218 (32.4%)	142 (38.8%)	
Distant metastasis						
No	586 (99.3%)	334 (98.8%)	*p* = 0.423	668 (99.4%)	360 (98.4%)	*p* = 0.100
Yes	4 (0.7%)	4 (1.2%)		4 (0.6%)	6 (1.6%)	
Cell differentiation						
well	94 (15.9%)	47 (13.9%)	*p* = 0.408	97 (14.4%)	59 (16.1%)	*p* = 0.468
Moderate/poor	496 (84.1%)	291 (86.1%)		575 (85.6%)	307 (83.9%)	

* *p* < 0.05; ^a^ OR (95% CI):1.534 (1.160–2.028); ^b^ OR (95% CI):1.320 (1.013–1.721).

## Data Availability

The data presented in this study are available on request from the corresponding author.
